# D3PG-Light: A Lightweight and Stable Resource Scheduling Framework for UAV-Integrated Sensing, Communication, and Computation Systems

**DOI:** 10.3390/s26061829

**Published:** 2026-03-13

**Authors:** Qing Cheng, Wenwen Wu, Yebo Zhou

**Affiliations:** College of Air Traffic Management, Civil Aviation Flight University of China, Chengdu 610000, China; deglechq@163.com (Q.C.); 13777722607@163.com (Y.Z.)

**Keywords:** integrated sensing, communication, and computation, unmanned aerial vehicle, deep reinforcement learning, mobile edge computing, 6G

## Abstract

Unmanned Aerial Vehicles (UAVs) are gradually emerging as key platforms for Integrated Sensing, Communication, and Computation (ISCC) systems in next-generation wireless networks. However, strict resource constraints and task coupling make static allocation inefficient in dynamic environments. This paper studies a UAV-driven ISCC system in which a single UAV dynamically allocates communication bandwidth, sensing resources, and computing power. Considering that sensing data in mission-critical applications is highly time-sensitive, minimizing the response time is paramount. To reduce system latency while maintaining sensing quality and energy efficiency, we propose D3PG-Light, a deployment oriented and stability-enhanced refinement of the deep reinforcement learning framework, specifically tailored for real-time resource scheduling under UAV hardware constraints. D3PG-Light incorporates an adaptive gradient stabilization mechanism, Long Short-Term Memory (LSTM), and feature fusion to enhance training stability. Simulation results based on real air–ground channel measurements show that D3PG-Light converges faster and achieves more stable learning behavior than DDPG, TD3, and the original D3PG. In particular, the proposed method reduces the 95th-percentile latency from over 100 ms to approximately 24 ms, achieves higher converged reward values, and requires fewer than 50 k model parameters. These results demonstrate the effectiveness of D3PG-Light for latency-sensitive UAV-ISCC applications.

## 1. Introduction

The sixth-generation (6G) era of mobile communications moves towards intelligent connectivity, requiring the seamless integration of sensing, communication, and computing (ISCC) to support ubiquitous services [1,2]. Within the ISCC framework, a unified resource pool and control policy are employed to jointly optimize these functionalities, which has been identified as a key research frontier for 6G [3,4,5]. Specifically, in low-altitude scenarios, UAVs operate under strict battery and payload constraints, while sensing, communication, and computation tasks compete for limited onboard resources, including communication bandwidth, computing frequency, and sensing time slots. Traditional static resource allocation strategies cannot adapt to the rapid changes in channel quality and task arrivals, leading to severe performance degradation. Therefore, investigating dynamic resource scheduling for a single UAV—the fundamental unit of aerial edge networks—is essential for ensuring QoS, particularly given UAVs’ three-dimensional mobility, rapid deployment capability, and reliable line-of-sight air-to-ground links in low-altitude ISCC scenarios. Focusing on a single UAV as the fundamental scheduling unit simplifies model complexity while enabling detailed analysis of real-time decision-making under hardware constraints. This aligns with recent works that demonstrate the tractability and practical value of single-agent DRL frameworks before scaling to multi-UAV systems [6,7].

However, achieving deep integration of communication, sensing, and computing functionalities on a UAV mobile platform still faces many challenges. First, regarding environment modeling and adaptability, traditional studies often adopt overly idealized assumptions that fail to reflect real low-altitude dynamics. Furthermore, sophisticated channel characterization is essential for capturing high-fidelity measurements in cognitive aerial networks [8]. Practical air-to-ground links are profoundly influenced by dynamic variations caused by UAV posture changes and fuselage scattering effects [9], which introduce additional stochasticity into scheduling decisions.

Second, there are clear gaps in the training stability and lightweight deployability of intelligent scheduling algorithms. In this work, training stability primarily refers to the convergence behavior of learning algorithms in high-dimensional continuous action spaces under non-stationary air–ground channel conditions. Meanwhile, lightweight deployability denotes constraints on model parameter scale, memory footprint, and inference latency on edge or onboard platforms. Deep reinforcement learning (DRL) has shown potential in UAV resource optimization because it can effectively solve high-dimensional, non-convex control problems in dynamic environments without requiring an explicit and precise mathematical model of the system. This is particularly critical because the limited onboard battery capacity of UAVs cannot sustain long-term high-intensity computation, and any inference delay beyond the millisecond level could lead to outdated scheduling decisions in highly dynamic environments. Accordingly, resource scheduling is formulated as a reinforcement learning problem in which the UAV acts as an agent that observes system states and outputs resource allocation actions guided by a designed reward function.

The fundamental motivation for resource scheduling in this work is to balance the inherent trade-offs between system latency, sensing accuracy, and energy consumption under strict UAV hardware constraints. While traditional optimization methods, such as convex optimization or Lyapunov-based techniques, have been used for resource allocation, they often struggle with the non-convex resource coupling and the lack of precise mathematical models in highly dynamic low-altitude environments [10,11,12]. The rationale for adopting deep reinforcement learning (DRL) in this study is its inherent capability to learn optimal policies through autonomous interaction without requiring an explicit mathematical model of the complex, non-stationary air-to-ground channels. Specifically, the D3PG-based framework is chosen to handle the high-dimensional continuous action space of ISCC while ensuring superior training stability and lightweight deployment [13,14]. To ensure real-time execution on resource-constrained UAVs, we further introduce a lightweight design to minimize inference latency. In summary, this paper proposes D3PG-Light, a unified and deployment-oriented scheduling framework designed to bridge the gap between theoretical DRL and practical UAV-ISCC operations. The main contribution of this work lies in the practical integration of high-fidelity environmental modeling with a stability-enhanced algorithm. Specifically, we leverage real-world air–ground channel measurements and queue dynamics to construct a realistic testbed. On this basis, D3PG-Light refines the classical deterministic policy gradient paradigm by incorporating adaptive gradient stabilization and temporal feature fusion. This synthesized approach allows for a significant reduction in model complexity—achieving a 73.05% parameter reduction compared to the original D3PG—while simultaneously suppressing 95th-percentile latency to 24 ms. The remainder of this paper is organized as follows. Section 2 reviews related work on algorithm-based and learning-based resource scheduling. Section 3 presents the system model and unified ISCC environment construction. Section 4 describes the proposed D3PG-Light framework in detail. Section 5 reports the experimental setup and performance evaluation results. Finally, Section 6 concludes the paper and outlines future research directions.

## 2. Related Work

The dynamic management of network resources has been a focal point of research in wireless communications and edge computing. The existing literature can be broadly categorized into algorithm-based and machine-learning-based approaches.

### 2.1. Algorithm-Based Resource Scheduling

Classical scheduling in UAV-enabled ISCC systems typically relies on mathematical programming and decomposition techniques. Many works formulate long-term scheduling problems and apply Lyapunov optimization to stabilize queues while minimizing system cost. For example, Lin et al. [15] transform a time-dependent cost minimization into a per-slot decision by introducing a Lyapunov function. In their Lyapunov-based scheme (LDRA), the long-term UAV resource allocation problem is converted into slot-level optimization problems, and a greedy matching algorithm is used to schedule tasks each time slot. Similarly, Dai et al. [16] formulate an online UAV-assisted offloading problem and decouple the long-term energy constraint based on Lyapunov optimization, which enables the scheduling to be solved in real-time without future knowledge. Beyond these specific UAV scenarios, the fundamental principles of resource management were established in early studies of complex network topologies. For instance, in multi-rate multi-channel mesh networks, joint optimization of channel assignment and rate adaptation was achieved through decomposition-based heuristic algorithms to manage cross-layer interference [17]. Subsequent research expanded these classical frameworks by incorporating robust optimization to handle link uncertainty and interference alignment to maximize spectral efficiency in multi-user interference channels [18].

To reduce complexity, many works employ heuristic or metaheuristic algorithms. Genetic Algorithms (GAs) have been used for joint offloading and trajectory optimization, trading optimality for faster execution [19,20,21,22]. For instance, Attalah et al. [23] propose a GA-based offloading scheme in a “hybrid fog” IoD architecture, using a GA to optimally offload UAV tasks to fog base stations and mobile fog UAVs, significantly reducing total delay. Likewise, particle swarm optimization (PSO) and its variants have been applied: Adaptive variants of PSO have also been explored. For example, Perera et al. [24] propose a reinforcement-learning-controlled adaptive PSO (APSO) framework for task offloading in edge computing systems, demonstrating improved latency and resource utilization performance. Other heuristics include simulating annealing for trajectory and scheduling and greedy or local-search methods for allocation. In addition to heuristics, matching theory and game-theoretic frameworks have been widely employed to coordinate the competitive resource sharing among multiple UAVs or users. For example, some studies leverage stable matching algorithms to solve task-offloading and sub-channel allocation problems, ensuring a balance between individual rationality and system-wide efficiency without excessive iterations [25]. Recently, for Integrated Sensing and Communication (ISAC) systems, non-ML optimization research has pivoted toward Semi-Definite Programming (SDP) and Successive Convex Approximation (SCA) to tackle the non-convex performance trade-offs between sensing mutual information and communication throughput [26]. While these methods provide mathematically rigorous bounds, their complexity scales cubically with the number of antennas or subcarriers, making them less practical for real-time UAV onboard execution. While heuristic and game-theoretic approaches are relatively lightweight and easy to implement, but they often lack global optimality guarantees and typically require offline parameter tuning.

In summary, algorithmic schedulers provide tractable solutions for UAV scheduling problems, but they generally rely on accurate models and can be slow or suboptimal when system dynamics are fast. Such methods often do not readily adapt to highly stochastic environments, motivating learning-based alternatives for online adaptation [27,28].

### 2.2. Machine-Learning-Based Resource Scheduling

Machine learning, particularly deep reinforcement learning (DRL), has been widely adopted to handle the dynamics and uncertainty of UAV-enabled ISCC systems. A variety of DRL architectures have been applied to learn scheduling policies from data rather than solving optimization from scratch. For example, Darchini-Tabrizi et al. [29] model the offloading problem in a multi-UAV MEC system as a Markov decision process and employ a Rainbow DQN to make task-offloading decisions. Their DRL agent quickly converges and achieves ~12.6% lower latency than state-of-the-art baselines. In another work, Li et al. [30] apply a Soft Actor–Critic (SAC)-based deep reinforcement learning approach to jointly learn computation offloading decisions and resource allocation in a UAV-assisted MEC setting, formulating the task-offloading policy as a Markov decision process.

Multi-agent reinforcement learning (MARL) has also been explored for coordinated UAV scheduling and task offloading. For instance, Zhu et al. [19] propose a multi-agent deep reinforcement learning framework that models multi-UAV trajectory and task offloading as a decentralized partially observable Markov decision process (Dec-POMDP), integrating the QTRAN algorithm with large language models (LLMs) and graph convolutional networks to efficiently capture inter-subregion relationships. Their approach shows significantly faster convergence and higher task completion rates compared with conventional DRL methods. Similarly, Ju et al. [31] investigate a multi-UAV assisted mobile edge computing system where each UAV is treated as an agent and apply the Multi-Agent Deep Deterministic Policy Gradient (MADDPG) algorithm to jointly optimize task-offloading strategies and UAV flight trajectories, reducing overall energy consumption and computation latency relative to baseline schemes. Other works use value-decomposition networks, MAPPO, or actor–critic networks to coordinate multiple UAVs, treating each UAV as an agent that learns to cooperatively offload tasks. Such MARL schemes can learn distributed policies under partial observability, but they suffer from high model complexity, extensive training time, and sensitivity to hyperparameters.

In general, ML-based schedulers deliver near-optimal, data-driven solutions without explicit modeling of all dynamics, but at the cost of heavy training and large inference cost. They may also lack theoretical guarantees and require large amounts of experience data. Moreover, while DRL-based schedulers can learn adaptive policies, most existing approaches involve large neural network models and high computational requirements, since they often prioritize policy performance over model compactness. When deployed on UAV or edge computing platforms with strict battery and computation constraints, large models may incur significant inference latency and memory overhead, which undermines real-time decision-making capabilities. This challenge has recently motivated research into lightweight model design and model compression techniques to alleviate training and inference costs on resource-constrained platforms [32]. While many DRL-based schedulers focus on communication and computation resources (e.g., offloading, power allocation, trajectory planning), few explicitly consider the generation, processing, and scheduling of sensing data as part of an Integrated Sensing, Communication, and Computing (ISCC) resource allocation framework. This lack of unified treatment overlooks the interdependencies among sensing accuracy, communication quality, and computing effort that are essential in ISCC scenarios [33]. These limitations motivate the development of lighter-weight DRL approaches. In this work, we propose a lightweight DRL scheduler that retains the adaptability of learning-based methods while reducing computational overhead, leveraging insights from both classical and ML-driven scheduling.

## 3. System Model and Problem Formulation

### 3.1. System and Resource Description

**Figure 1:**
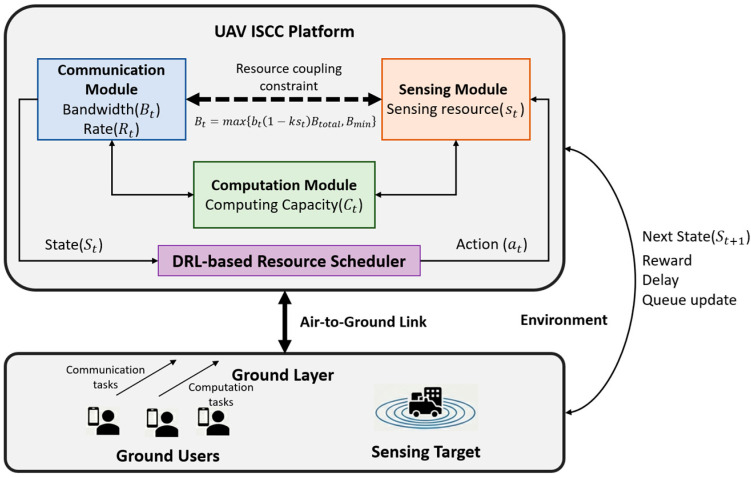
Single-UAV ISCC system architecture.

We consider a fully mobile single UAV acting as a low-altitude ISCC platform serving ground users. The UAV moves in a three-dimensional (3D) space to dynamically adjust its proximity to ground targets and users, thereby optimizing sensing and communication performance. The overall system architecture is illustrated in Figure 1, which comprises three functional modules: communication, sensing, and computation. These modules share the limited onboard resources and are dynamically controlled by a deep reinforcement learning (DRL) agent, and the agent observes the system state and executes resource allocation actions.

The system operates in discrete time slots indexed by t. In each slot, the UAV’s spatial position is updated based on its velocity and heading, ensuring a continuous flight trajectory rather than hovering at fixed coordinates. Specifically, the resource coupling constraint implies that sensing activities occupy spectrum resources, thereby affecting communication bandwidth. In each time slot, ground users have communication data and computation tasks arriving modeled as Poisson processes, which enter the communication queue and computation queue maintained at the UAV [34]. The UAV agent selects a continuous action vector each slot:

(1) $$a_{t}=(b_{t},s_{t},c_{t}),$$

where $b_{t},s_{t},c_{t}\in \left[0,1\right]$ represent the normalized allocation fractions for three distinct resource dimensions: $b_{t}$ is the fraction of total communication bandwidth; $s_{t}$ is the fraction of time allocated to sensing within each scheduling slot ∆t. Specifically, we adopt a Time-Division Multiplexing (TDM) approach where $s_{t}∆t$ is the duration dedicated to radar pulse emission and echo reception. This allocation directly influences the sensing integration gain and the resulting processing delay; $c_{t}$ is the fraction of the UAV’s maximum computing frequency allocated for task execution; let $F_{max}$ denote the maximum CPU clock frequency of the UAV’s onboard processor. The allocated computing frequency at slot t is $f_{t}=c_{t}\;F_{max}$. This frequency is then converted into computing capacity $C_{t}$ by $C_{t}=f_{t}/\omega$, where ω represents the required CPU cycles per floating-point operation. Specifically, the term $ks_{t}$ in Equation (2) represents the bandwidth reduction factor, where k=0.15 is a linear coupling coefficient indicating that for every unit of resource allocated to sensing, a proportional amount of bandwidth is unavailable for communication [35,36,37]. Thus, the actual communication bandwidth in slot t is given by

(2) $$B_{t}=max\left\{b_{t}\left(1-ks_{t}\right)B_{total},B_{min}\right\},$$

where $B_{total}=5\;MHz$ is the total system bandwidth and $B_{min}=10\;kHz$ is a minimum bandwidth floor to avoid dropping below a safe threshold.

The UAV communicates with users over an air-to-ground wireless link. While our analysis prioritizes time-delay factors, it is crucial to recognize that the underlying propagation environment involves complex physical phenomena, such as posture-dependent shadowing and fuselage scattering. These channel characteristics, often characterized via advanced sounding systems, underscore the necessity of adaptive control frameworks like DRL to handle environmental non-stationarity [8,9]. In this work, the air–ground channel dynamics are driven by a large-scale measured envelope dataset, comprising 7534 independent channel scenarios with 9604 temporal samples per scenario, over 72 million samples in total. The dataset contains normalized channel envelope values within [0, 1], with mean 0.269 and standard deviation 0.310, reflecting significant small-scale fading variability. Statistical inspection shows no NaN or infinite values, and less than 0.3% show zero entries, indicating data completeness and stability. These temporally aligned channel traces are directly incorporated into the simulation environment to reproduce realistic non-stationary air–ground fading behavior during both training and evaluation phases. According to Shannon’s formula, or allocated bandwidth $B_{t}$ and instantaneous signal-to-noise ratio ${SNR}_{t}$, the downlink data rate is

(3) $$R_{t}=B_{t}\log_{2}\left(1+{SNR}_{t}\right)\;\left(\frac{bits}{second}\right),$$

**Figure 2:**
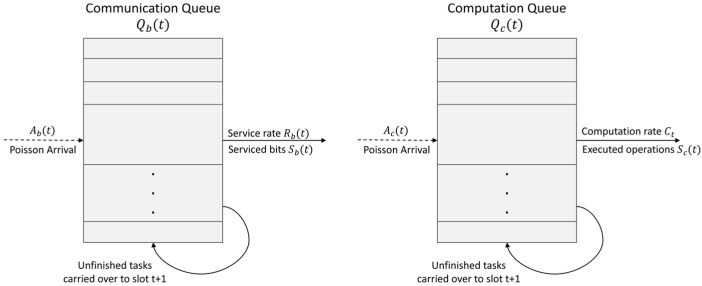
Arrival-service dynamics in ISCC systems. The dots indicate omitted queued tasks for visual clarity.

Using the available communication rate $R_{t}$ and computing capacity $C_{t}$, the UAV serves the queued tasks. As shown in Figure 2, the service process removes tasks from the queue based on the allocated resources. Let $S_{b}\left(t\right)$ and $S_{c}\left(t\right)$ denote the actual amount of data bits and computing operations processed in slot t, which are determined by $S_{b}\left(t\right)=min\{Q_{b}\left(t\right),R_{t}\Delta_{t}\}$ and $S_{c}\;\left(t\right)=min\{Q_{c}\;\left(t\right),C_{t}\Delta_{t}\}$, respectively, where τ is the slot duration. Consequently, the queues evolve as

(4) $$Q_{b}\left(t+1\right)=Q_{b}\left(t\right)+A_{b}\left(t\right)-S_{b}\left(t\right),Q_{c}\left(t+1\right)=Q_{c}\left(t\right)+A_{c}\left(t\right)-S_{c}\left(t\right),$$

where $Q_{b}\left(t\right)$ and $Q_{c}\left(t\right)$ are the backlog lengths of the communication and computation queues at the beginning of slot t, and $A_{b}\left(t\right)$ and $A_{c}\left(t\right)$ are the amounts of newly arrived communication data and computing tasks during slot t, respectively. Through the above closed-loop process, the UAV’s action influences the next slot’s state, realizing a sensing–decision–feedback control loop.

To support the agent’s decision-making, we define the system state vector as

(5) $$S_{t}=\left[\mu_{g},\sigma_{g},g_{min},g_{max},{SNR}_{t},d_{norm},h_{norm},Q_{b,norm},Q_{c,norm}\right],$$

where $\mu_{g}$, $\sigma_{g}$, $g_{min}$, $g_{max}$ denote the recent channel mean, standard deviation, minimum, and maximum; ${SNR}_{t}$ is the current signal-to-noise ratio; $d_{norm}$ and $h_{norm}$ are the normalized horizontal distance and altitude of the UAV relative to the ground center, which vary over time as the UAV flies. $Q_{b,norm}$, $Q_{c,norm}$ are the normalized queue lengths. This state design, together with protections such as $B_{min}$, a noise lower bound, and an SNR upper bound, ensures the learning agent receives stable and meaningful observation at every step, avoids out-of-range values that could destabilize training, and captures the information necessary for decision-making.

### 3.2. Delay Model

To evaluate the latency of each type of task, we decompose the delay into inherent processing time and queue waiting time. This avoids the unrealistic situation of zero delay when a queue is empty. To maintain numerical consistency with the simulation time slot, all delay values calculated in seconds are scaled by a factor of 1000 to represent milliseconds before being incorporated into the reward function.

#### 3.2.1. Communication Delay

We assume each transmission involves a data packet of nominal length L bits. At a rate $R_{t}$, the inherent transmission time for one packet is $L/R_{t}$. If the communication queue has $Q_{b}(t)$ bits waiting at the start of slot, the additional waiting time required to clear this backlog at the current rate is $Q_{b}(t)/R_{t}$. Therefore, the communication delay in slot t is modeled as

(6) $$D_{comm,t}=\frac{L}{R_{t}}+\frac{Q_{b}\left(t\right)}{R_{t}},$$

#### 3.2.2. Computation Delay

Similarly, for computation tasks, let the nominal task size be M floating-point operations. At a computing rate denoted by $C_{t}=max\{c_{t}C_{max},C_{min}\}$, where $C_{max}=F_{max}/\omega$ is the peak processing capacity derived from the maximum CPU frequency. Here, $C_{min}$ represents the minimum processing floor to maintain basic system functions, respectively. The inherent execution time for a task is $M/C_{t}$. If $Q_{c}\left(t\right)$ operations remain in the computation queue, the waiting time is $Q_{c}\left(t\right)/C_{t}$. Thus, the computation delay is

(7) $$D_{comp,t}=\frac{M}{C_{t}}+\frac{Q_{c}\left(t\right)}{C_{t}},$$

#### 3.2.3. Sensing Delay

In each time slot, the UAV may perform a sensing task. We assume sensing tasks are periodic and do not queue; however, their duration depends on the allocated sensing resource fraction and the current channel quality. Specifically, within each scheduling slot T, the fraction $s_{t}$ represents the time dedicated to active radar integration. A larger $s_{t}$ increases the signal energy accumulation, effectively accelerating the target detection/recognition process. Let $T_{s}^{base}$ be a baseline sensing period under minimum resource allocation and poor SNR conditions. Allocating a sensing resource fraction $s_{t}$ accelerates the sensing process, and a higher communication ${SNR}_{t}$ further improves sensing efficiency. We model the sensing delay as

(8) $$D_{sense,t}=\frac{T_{s}^{base}}{\left(1+\eta {SNR}_{t}\right)\left(1+s_{t}\right)},$$

where η is an SNR gain factor. We use η=0.8, representing the contribution of a positive SNR to reducing sensing time. In this model, allocating more sensing resources and having a higher SNR both reduce the sensing delay, but with diminishing marginal benefit.

The total system delay in slot t is the sum of the above three components:

(9) $$D_{tot,t}=D_{comm,t}+D_{comp,t}+D_{sense,t},$$

In implementation, to ensure numerical stability, we convert all delay values to milliseconds and set a small positive lower bound to avoid extreme cases of zero delay.

### 3.3. Reward Function Design

The immediate reward is carefully designed to balance three main objectives: low latency, high sensing accuracy, and energy efficiency. Given the non-convex nature of the multi-resource coupling in ISCC and the difficulty of obtaining an analytical optimal solution in dynamic environments, we construct a multi-objective reward function to guide the DRL agent’s exploration. We integrate these goals into a single reward with weighted terms and include penalty terms to discourage undesirable behavior. We note that the reward design is heuristic in nature, which is common in practical DRL-based resource scheduling [38,39]. Our focus is on empirical stability and performance rather than analytical optimality. The piecewise combined reward for each slot t is given by

(10) $$R_{t}=-w_{d}\Phi \left(D_{tot,t}\right)+w_{a}A_{s,t}+w_{e}E_{c,t}-\lambda_{r}\left(b_{t}+s_{t}+c_{t}\right)-\lambda_{E}E_{tot,t}-P_{ext,t}-P_{sm,t}$$

in this reward structure, $w_{d}$, $w_{a}$, and $w_{e}$ are positive weighting coefficients for delay, sensing accuracy, and energy consumption, respectively. The selection of weighting coefficients follows a structured two-stage approach to ensure design confidence and optimization stability. First, we apply magnitude normalization, where each coefficient is inversely proportional to the expected numerical range of its corresponding reward component. For instance, since the delay penalty $\Phi \left(D_{tot,t}\right)$ can span several orders of magnitude, its weight is carefully scaled to prevent it from overwhelming the gradients of the sensing accuracy and energy terms during the early exploration phase. Second, the final values are determined based on mission-oriented priorities. In our UAV-ISCC scenarios, $w_{d}$ is prioritized and set to 1.0 to emphasize that satisfying strict latency constraints is paramount for flight safety and mission timing. The weights for sensing and energy efficiency are then assigned to achieve a Pareto-optimal balance, as confirmed by the sensitivity analysis in Section 5.4. The term $A_{s,t}=1-e^{-2s_{t}(1+{SNR}_{t})}$ denotes the normalized sensing accuracy, while $E_{c,t}=\ln\left(1+\frac{S_{b}\left(t\right)}{E\left[Q_{b}\right]}\right)$ represents the task processing efficiency. The final two terms, $P_{ext,t}$ and $P_{sm,t}$, denote the cascaded extreme penalty and the action smoothing penalty, respectively, to ensure system stability. Here, the individual components are defined as follows:

1. Total delay penalty: We impose a penalty on the total latency, using a log compression, $\Phi (D_{tot,t})=log(1+D_{tot,t}/100)$, where 100 is a normalization scale to keep delays comparable and $w_{d}$ is the weight for the delay term.
2. Sensing accuracy reward: Better sensing performance is achieved by allocating more sensing resources under good channel conditions. We introduce an accuracy term that rewards the agent for allocating sensing resources when the channel is favorable. This term is designed to saturate as it approaches 1, diminishing returns for very high resource allocation or SNR. We add this accuracy term to the reward with a positive weight $w_{a}$.
3. Communication efficiency reward: To encourage efficient use of communication resources, we include an efficiency term defined as the log of the fraction of incoming data successfully transmitted. Specifically, where $S_{b}(t)$ is the amount of data (bits) transmitted in slot t and $E\left[Q_{b}\right]=\lambda_{b}\Delta t$ is the data arrival in that slot. This term is weighted by $w_{e}$.
4. Resource usage penalty: We apply a small penalty proportional to the total fraction of resources used. The purpose is to discourage the agent from always pushing all resources to their maximum limits. $\lambda_{r}$ is a small penalty coefficient.
5. Energy consumption penalty: To promote sustainable operation, we incorporate an energy-aware penalty term into the reward structure. The total energy consumption of the UAV-ISCC platform in slot t is modeled as the summation of the hardware overhead from communication, computation, and active sensing modules:
  (11) $$E_{tot,t}=E_{comm,t}+E_{comp,t}+E_{sense,t}=\left(P_{tx,t}+\kappa {\left(c_{t}f_{max}\right)}^{3}+P_{sen}\cdot s_{t}\right)\cdot \Delta t,$$
  where $b_{t}$, $c_{t}$, and $s_{t}$ denote the normalized allocation fractions for communication bandwidth, computing, and sensing, respectively, and Δt is the slot duration. $P_{tx,t}$ denotes the transmit power in slot t, defined as $P_{tx,t}=P_{tx}^{max}b_{t}^{\alpha }$, where $P_{tx}^{max}$ is the maximum transmit power and α is the bandwidth–power coupling exponent. $P_{sen}$ denotes the sensing-module power coefficient. The term $\kappa (c_{t}f_{max}{)}^{3}$ models the dynamic power consumption of the onboard CPU under a DVFS-based model, where κ is the effective switched-capacitance coefficient and $f_{max}$ is the peak CPU operating frequency. This holistic energy model captures the multi-dimensional hardware costs, encouraging the DRL agent to optimize resource allocation while avoiding excessive power depletion.
6. Cascaded extreme penalty $P_{ext,t}$: To prevent the agent from entering danger zones where both latency and energy consumption spike beyond system tolerances, we introduce a threshold-based cascaded penalty:
  (12) $$P_{ext,t}=\left\{\begin{array}{cc} 0.5,\; & D_{tot,t}>500\;and\;b_{t}+s_{t}+c_{t}>1.5\\ 0.3,\; & D_{tot,t}>300\;and\;\lambda_{E}E_{tot,t}>0.1\\ 0.4,\; & D_{tot,t}>800\\ \;\;\;0, & otherwise \end{array}\right.,$$
  where $D_{t}$ is the total latency in milliseconds, $E_{tot,t}$ is the slot energy consumption, and $\lambda_{E}$ is the energy normalization coefficient used in the reward. This piecewise design provides strong corrective signals only when the system approaches unsafe operating regimes, thereby improving robustness while avoiding overly restrictive penalties in normal operating conditions.
7. Action smoothing penalty $P_{sm,t}$: To suppress high-frequency mechanical oscillations and ensure stable transitions between scheduling decisions, a smoothing penalty is imposed on the action variation:
  (13) $$P_{sm,t}=\lambda_{ext}\cdot {\left\|a_{t}-a_{t-1}\right\|}^{2},$$
  where $a_{t}=\left(b_{t},s_{t},c_{t}\right)$ denotes the resource allocation vector at slot t. This term encourages the learned policy to maintain temporal continuity, which is essential for preserving the lifespan of UAV onboard actuators. The smoothing coefficient $\lambda_{s}$ is chosen to be sufficiently small so that it regularizes extreme oscillations without dominating the primary optimization objective.

### 3.4. MDP Formulation and Problem Description

**Figure 3:**
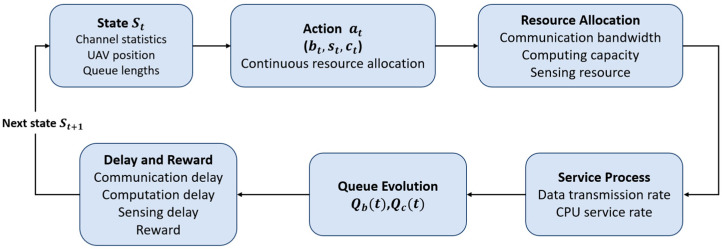
Schematic of the MDP decision-making loop.

We formulate the UAV’s resource scheduling as a continuous-state, continuous action Markov decision process (MDP). The overall decision-making process is visualized in Figure 3. At each time step, the agent observes the state vector $S_{t}$ and outputs a resource allocation action $a_{t}=(b_{t},s_{t},c_{t})$. The environment then computes the system latency and energy consumption, transitions to a new state $S_{t+1}$ according to the queue dynamics and channel evolution, and returns an immediate reward $P_{s,t}$.

The objective of the agent is to find an optimal policy $\Pi^{*}$ that maximizes the long-term expected return:

(14) $${max}_{\pi }\;E_{\pi }[\sum_{t=0}^{\infty }\gamma^{t}\;R_{t}],$$

where γ∈(0,1) is the discount factor. By solving this MDP, the agent can approach the optimal policy that maximizes the cumulative reward in the dynamic environment, achieving an optimal trade-off among multiple performance metrics such as communication throughput, computation delay, and sensing accuracy.

## 4. Improved D3PG-Light Algorithm Design and Implementation

### 4.1. Design Goals and Overview

This problem falls under continuous-state, continuous action reinforcement learning, with a nine-dimensional continuous-state space and a three-dimensional continuous action space, already constrained by the environment to ensure physical feasibility. In light of the training instability and value estimation bias issues that standard DDPG can encounter in high-dimensional, non-stationary environments, we propose a D3PG-Light algorithm. D3PG-Light builds upon DDPG with several enhancements, aiming to improve stability and performance while keeping the model lightweight [40,41].

The main improvements include the following:

1. Optimized neural network capacity: We adopt an appropriately sized network architecture and apply layer normalization at each layer output to enhance representation capability for high-dimensional state features while suppressing gradient explosion.
2. Innovative Feature Fusion (IFF) module: Considering the heterogeneity of the state vector, which consists of channel-related features and queue-related features, we design specialized sub-networks to process each part separately and then fuse them at a higher level, enhancing the ability to jointly perceive different categories of information.
3. Adaptive Gradient Stabilization (AGS) mechanisms: We employ a series of gradient stabilization strategies to ensure numerical stability during training and reduce the risk of gradient explosion or divergence.

In summary, the goal of D3PG-Light is to achieve stable convergence in training and high-performance decision-making for the continuous control task in a complex UAV environment, while keeping the model lightweight for efficient onboard inference. It is worth noting that D3PG-Light is not intended to propose a fundamentally new RL paradigm, but a stability-oriented and deployment-aware refinement of deterministic policy gradient methods tailored for UAV-ISCC systems.

### 4.2. Neural Network Architecture Design

**Figure 4:**
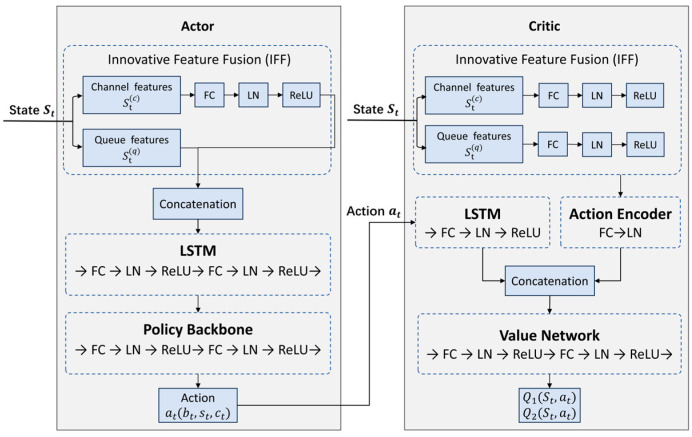
Neural network architecture of the proposed D3PG-Light algorithm.

D3PG-Light adopts a modular actor–critic network structure designed to exploit the heterogeneous nature of state features while ensuring stable gradient propagation. The overall network architecture is illustrated in Figure 4. Both the actor and critic networks are composed of multiple fully connected (FC) layers, an Integrated Feature Fusion (IFF) module, and LSTM units.

#### 4.2.1. Innovative Feature Fusion (IFF) Module

Given that the state vector S_t is composed of features from different categories, we introduce an Innovative Feature Fusion (IFF) module. This module processes channel-related features and queue-related features through separate embedding networks before fusion. We can split the state into two parts: $S_{t}=\left[S_{t}^{\left(c\right)},S_{t}^{\left(q\right)}\right]$, where $S_{t}^{\left(c\right)}$ includes channel and link quality features and $S_{t}^{\left(q\right)}$ includes queue lengths and delay indicators.

IFF processes each part to extract effective embeddings with distinct strategies. To ensure balanced feature representation, the channel and queue features are processed independently through parallel branches. Each branch consists of a fully connected (FC) layer, followed by layer normalization (LN) and a ReLU activation function. Subsequently, these refined channel and queue embeddings are concatenated to form the unified state representation. The IFF module allows the network to both distinguish and jointly utilize the two types of information at higher layers, while the use of layer normalization mitigates training instability due to differences in feature scale. In our ablation experiments, we will show that the IFF module significantly improves policy learning performance.

#### 4.2.2. Actor Network Design

The actor network in D3PG-Light is used to approximate the deterministic policy $\mu_{\theta }(s)$. It takes state S as an input and outputs an action $a_{t}$. The structure is as follows: first, the input state is processed by the IFF module described above to extract and fuse features, yielding a hidden representation of dimension H. Then, an LSTM layer is employed to process this representation for temporal modeling. This hidden vector is then passed into the policy backbone network. In our design, the policy backbone consists of two hidden layers with adaptive gradient stabilization (AGS) features: each hidden layer is a fully connected (FC) linear layer followed by layer normalization and a ReLU activation. Such linear layers with layer norm can constrain activations and gradient norms to some extent, serving a similar role as the BatchNorm used in the original DDPG paper, but layer norm is more suitable for time-dependent or non-i.i.d. inputs, thus working better in our sequential decision scenario.

It should be noted that we implement the actor network in a lightweight fashion: the hidden layers have relatively small widths (e.g., 64 and 128), resulting in a total parameter count of around 48 k, thereby ensuring efficient inference. Despite the modest size, with feature fusion and normalization, the network retains strong representational power.

#### 4.2.3. Critic Network Design

To mitigate the overestimation bias inherent in standard DDPG, the critic module in D3PG-Light employs two independent critic networks to implement the clipped double Q-learning mechanism. During the update, the target value is calculated as the minimum of the two target critic outputs. The critic network is used to estimate the Q-value $Q_{\Phi }(s,a)$. Its structure is similar to the actor’s: the state S is first passed through an independent IFF module to obtain a state embedding. Simultaneously, the action $a_{t}$ is passed through a fully connected (FC) linear layer with layer normalization to obtain an action embedding. As illustrated in Figure 4, the state embedding from the IFF and LSTM and the action embedding from the Action Encoder are concatenated into a joint feature vector before being passed into the subsequent value network for Q-value estimation. The value network contains two fully connected layers, each followed by ReLU and layer normalization, and finally outputs a linear Q-value estimate. To prevent Q-value overestimation, we clamp the critic’s output to a certain range and train using the Huber loss, which is more robust to outliers than mean squared error (MSE). The heavy use of layer normalization, FC layers, and IFF in the critic network is crucial for training stability: if these design elements are removed, we observed a significant drop in learning performance.

#### 4.2.4. LSTM Extension for Temporal Features

For scenarios with strong temporal correlations, D3PG-Light supports extending the actor and critic networks to include recurrent units, specifically a Long Short-Term Memory (LSTM) layer, to model historical information [40,41,42]. With the LSTM extension, the agent takes the sequence of recent N states as input to capture temporal evolution of the state. We insert a single-layer LSTM between the IFF module and the subsequent fully connected layers, feeding the sequence of per-slot features extracted by IFF into the LSTM. The LSTM hidden layer size is 64, and the hidden state is reset at the beginning of each new episode. At each decision step, the LSTM processes the new input along with its previous hidden state to produce a new hidden state, which is then passed to the following network layers. During training, we maintain the continuity of the LSTM hidden states for both actor and critic, and use truncated backpropagation through time when sampling sequences from the replay buffer for efficiency.

### 4.3. Adaptive Gradient Stabilization Mechanism

To ensure stable reinforcement learning training, D3PG-Light integrates an adaptive gradient stabilization (AGS) mechanism. AGS comprises multiple strategies to suppress gradient divergence and mitigate learning instability, including the following:

1. Gradient norm clipping: When updating the actor or critic network parameters, we impose an upper threshold on the gradient norm. If the norm exceeds 1.0, we clip it to that maximum. This hard clipping prevents occasional gradient spikes from destabilizing the network’s convergence.
2. Exploration noise scheduling: We combine different types of noise to improve exploration efficiency. D3PG-Light uses a two-stage noise decay strategy: in early training, we use Ornstein–Uhlenbeck (OU) noise with temporal correlation for exploration; as training progresses, the OU noise is gradually reduced and we switch to Gaussian noise in later stages. We can also experiment with Beta-distributed noise to enhance stable boundary exploration. By dynamically adjusting the noise type and intensity across training stages, the agent can explore effectively while avoiding excessive oscillation.
3. Target network soft update: For both the actor and critic, we maintain a set of target network parameters $\left(\theta^{\mu^{\prime }},\theta^{Q^{\prime }}\right)$ that slowly track the learned network parameters. Specifically, after each update, we perform
  (15) $$\begin{array}{c} \theta_{Q_{j}}^{\prime }\leftarrow \tau \theta_{Q_{j}}+\left(1-\tau \right)\theta_{Q_{j}}^{\prime }\\ \theta_{\mu }^{\prime }\leftarrow \tau \theta_{\mu }+\left(1-\tau \right)\theta_{\mu }^{\prime }\;\;\;j\epsilon \left\{1,2\right\}, \end{array}$$
  where 0<τ≤1 is the soft-update coefficient; we typically use τ=0.01.

These measures collectively form the AGS mechanism of D3PG-Light, aligned with strategies in other domains for improving RL stability. For example, combining Lyapunov optimization with PPO has been used to ensure queue stability in UAV-MEC systems. Similarly, in our high-dimensional stochastic environment, the AGS mechanism keeps the agent’s training process under control, significantly reducing occurrences of unstable oscillations or divergence.

### 4.4. Training Procedure and Implementation Details

Combining the above network structures and algorithmic enhancements, D3PG-Light follows a deterministic policy gradient framework with twin critics and clipped double Q-learning, which extends the classical DDPG paradigm for improved stability. Specifically, we adopt clipped double Q-learning with twin critics, Huber regression for critic updates, target policy smoothing, and delayed policy updates to improve training stability under non-stationary UAV-ISCC dynamics.

After each environment interaction step, we check if enough samples are in the replay buffer to perform a network update. For each update, the critic’s loss function is defined as

(16) $$L_{critic}=\frac{1}{N}\sum_{i=1}^{N}\sum_{j=1}^{2}Huber(Q_{j}\left({\tilde{S}}_{i},a_{i}\right)|\theta_{Q_{j}}-y_{i}),$$

where $y_{i}$ is the target Q-value for sample i. The target Q-value is given by

(17) $$y_{i}=R_{i}+\gamma \left(1-d_{i}\right){min}_{j=1,2}Q_{j}^{\prime }\left({\tilde{S}}_{i}^{\prime },{\tilde{a}}_{i}^{\prime }\right)$$

where γ=0.99 denotes the discount factor, and $d_{i}\in \left\{0,1\right\}$ is an indicator variable representing whether the terminal state has been reached: $d_{i}=1$ signifies termination, while $d_{i}=0$ indicates otherwise. Additionally, ${\tilde{a}}_{i}^{\prime }=Clip\left(\mu^{\prime }\left({\tilde{S}}_{i}^{\prime }\right)+\epsilon ,a_{min},a_{max}\right)$ is the target action with smoothed noise, and ϵ denotes the target policy smoothing noise.

Subsequently, for every critic update, the actor network is updated according to a predefined delayed policy update frequency. The actor update employs the policy gradient ascent method, where the policy parameters θ are adjusted to maximize the expected Q-value as estimated by the critic. Formally, the policy optimization objective is defined as

(18) $$L_{actor}\left(\theta_{\mu }\right)=-J_{actor}\left(\theta_{\mu }\right)=-\frac{1}{N}\sum_{i=1}^{N}Q_{1}\left({\tilde{S}}_{i},\mu ({\tilde{S}}_{i})\right),$$

Specifically, the objective is to optimize the actor network such that it generates actions that maximize the evaluated Q-value for a given state S. Based on the modular network architecture and the stabilization mechanisms discussed in the preceding sections, the overall training procedure of the D3PG-Light framework is synthesized into a structured reinforcement learning process. The detailed execution steps of the proposed D3PG-Light training process are summarized in Algorithm 1.

**Algorithm 1.** Training Procedure of D3PG-Light Framework**Input:** UAV-ISCC environment *ε*; number of training episodes *E*; maximum steps per episode *T*; discount factor *γ*; soft-update coefficient *τ*; batch size *N*; sequence length *L*; policy update delay *d*; actor and critic learning rates *ημ* and *ηQ*; gradient clipping threshold *C_clip_*; action bounds [*a_min_*,*a_max_*].
**Initialization:**

1:  Initialize the actor network
$\mu (\cdot |\theta_{\mu })$ with the IFF module and LSTM structure.
2:  Initialize the critic networks
$Q_{1}(\cdot |\theta_{Q_{1}}),Q_{2}(\cdot |\theta_{Q_{2}}).$

3:  Initialize the target networks by copying parameters:
$\theta_{\mu }^{\prime }\leftarrow \theta_{\mu },\;\theta_{Q_{j}}^{\prime }\leftarrow \theta_{Q_{j}},j\in \left\{1,2\right\}.$
4: Initialize the replay buffer *D* with capacity 10^5^.5: Initialize the exploration noise scheduler *N_t_*6: Set the global training step counter *t_global_* ← 0.
**Training Loop:**
7: For episode *e* = 1 to *E*, perform the following:
8:   Reset the environment *ε* and obtain the initial state
$S_{1}$.

9:   Initialize the LSTM hidden states
$h_{0}$ for the actor and critic networks.10: For step *t* = 1 to *T*, perform the following:
11:   Increment the global step counter:
$t_{global}\;\leftarrow \;t_{global}$ + 1.
12:   Embed the current state using the IFF module:
${\tilde{S}}_{t}=IFF(S_{t})$.
13:   Generate a deterministic action using the actor network:
$a_{t}=\mu ({\tilde{S}}_{t},h_{t-1}|\theta_{\mu }).$
14:   Sample exploration noise *n_t_* from
$N_{t}.$
15:   Apply exploration and clip the action:
$a_{t}\leftarrow Clip(a_{t}+n_{t},a_{min},a_{max}).$

16:   Execute action *a_t_* in the environment
ε.
17:   Observe the reward
$R_{t},\;\mathrm{next}\;\mathrm{state}\;S_{t+1},\;\mathrm{and}\;\mathrm{done}\;\mathrm{flag}\;d_{t}.$
18:   Store the transition
$\left(S_{t},a_{t},R_{t},S_{t+1},d_{t}\right)\;\mathrm{into}\;\mathrm{the}\;\mathrm{replay}\;\mathrm{buffer}\;D.$
19:   If the size of *D* 
≥N, then
20:     Randomly sample *N* state–action sequences of length *L* from
D.
21:     For each sampled sequence
i:
22:       Embed the next state using IFF:
${\tilde{S}}_{i}^{\prime }=IFF(S_{i}^{\prime })$.
23:       Compute the target action using the target actor:


$\;\;\;\;\;\;\;\;\;\;\;\;\;\;\;\;\;\;\;\;\;\;\;\;\;\;\;\;\;\;\;{\tilde{a}}_{i}^{\prime }=Clip\left(\mu^{\prime }\left({\tilde{S}}_{i}^{\prime }\right)+\epsilon ,a_{min},a_{max}\right).$


24:       Compute the target Q-value with clipped Double Q-learning (Equation (17)):


$\;\;\;\;\;\;\;y_{i}=R_{i}+\gamma (1-d_{i}){min}_{j=1,2}Q_{j}^{\prime }({\tilde{S}}_{i}^{\prime },{\tilde{a}}_{i}^{\prime }).$


25:      Update the critic networks by minimizing the Huber loss (Equation (16)):
$\;\;\;\;\;\;\;L_{critic}=\frac{1}{N}\sum_{i=1}^{N}\sum_{j=1}^{2}Huber(Q_{j}\left({\tilde{S}}_{i},a_{i}\right)-y_{i})$.
26:      Update critic parameters using gradient descent with gradient clipping:


$\;\;\;\;\;\;\;\theta_{Q_{j}}\leftarrow \theta_{Q_{j}}-\eta_{Q}\cdot ClipGrad(\nabla_{\theta_{Q_{j}}}L_{critic},C_{clip}),\;\;\;j\in \left\{1,2\right\}.$


27:
$\;\;\;\;\;\;\mathrm{If}\;(t_{global}\;\mathrm{mod}\;d==0),\;\mathrm{then}$
28:          Update the actor network by maximizing the expected Q-value (Equation (18)):
$\;\;\;\;\;\;\;J_{actor}\left(\theta_{\mu }\right)=\frac{1}{N}\sum_{i=1}^{N}Q_{1}\left(S_{i},\mu (S_{i})\right)$.
29:         Update actor parameters with gradient clipping:


$\;\;\;\;\;\;\;\theta_{\mu }\leftarrow \theta_{\mu }+\eta_{\mu }\cdot ClipGrad(\nabla_{\theta_{\mu }}J_{actor},C_{clip}).$


30:         Soft-update the target networks (Equation (15)):
$\;\;\;\;\;\;\;\theta_{\mu }^{\prime }\leftarrow \tau \theta_{\mu }+\left(1-\tau \right)\theta_{\mu }^{\prime }$,

$\;\;\;\;\;\;\;\theta_{Q_{j}}^{\prime }\leftarrow \tau \theta_{Q_{j}}+\left(1-\tau \right)\theta_{Q_{j}}^{\prime }.$


31:      End if.

32: End if.

33: Update the current state:
$S_{t}\leftarrow S_{t+1}$.
34: 
If d=1, then break.
35:  End for.
36: End for.
**Output**
Trained actor policy *μ*(*S*|*θμ*).

The above training loop covers data collection through environment interaction, network updates using experience replay, policy improvement, and target network synchronization, forming a complete closed loop. Through the specialized network architecture and multi-pronged stabilization strategies, the D3PG-Light algorithm achieves stable and efficient reinforcement learning training in the complex coupled UAV-ISCC environment, improving decision performance while ensuring stable convergence.

## 5. Experiments and Results Analysis

In this section, we evaluate the proposed D3PG-Light algorithm in the simulation environment and compare it with representative reinforcement learning baselines. To assess whether the framework fulfills the multi-objective operational requirements of UAV-ISCC systems—specifically balancing real-time response, task reliability, and hardware constraints—we select the following metrics as our primary evaluation criteria: (1) average reward to evaluate training stability; (2) system latency to ensure flight safety and mission timing; (3) sensing accuracy to measure task effectiveness; (4) energy consumption and model complexity to verify operational endurance and deployment feasibility on resource-constrained devices. These metrics are selected because they directly correspond to the three design goals of this work, namely stable policy learning, low-latency and sensing-effective resource scheduling, and lightweight deployment on resource-constrained UAV platforms. Therefore, jointly evaluating these metrics allows us to assess not only algorithmic performance but also practical feasibility for UAV-ISCC applications.

### 5.1. Experiment Setup and Environment Description

We use the single-UAV ISCC system simulation environment constructed in Section 2. The UAV acts as an airborne edge node, and each decision time slot involves resource allocation for communication, computation, and sensing, along with queue dynamics. Environment parameters such as total bandwidth, CPU peak computing power, task arrival rates, etc., are set to default values (see Table 1 for reference). Each training run lasts for 1000 episodes, with each episode containing up to 200 steps. The environment computes various costs according to Equations (1)–(11) and provides the corresponding reward. To reduce the impact of randomness, each experiment configuration is run five times with different random seeds, and we report the average of the key metrics for comparison.

To verify the algorithm’s real-time feasibility, we deploy the trained D3PG-Light model on an NVIDIA Jetson Orin Nano Super Developer Kit. This embedded edge AI platform features a 6-core Arm Cortex-A78AE v8.2 64-bit CPU and a 1024-core NVIDIA Ampere architecture GPU with 32 Tensor Cores, delivering up to 67 TOPS of AI performance. It is equipped with 8 GB of 128-bit LPDDR5 memory providing 102 GB/s bandwidth, which effectively supports the inference requirements of our lightweight neural networks. Regarding the software implementation, the simulation environment was built and trained on a general PC workstation running the Windows 11 operating system, using Python 3.12 and the PyTorch 2.7.0 deep learning framework. The trained models were subsequently deployed on the NVIDIA Jetson Orin Nano to verify real-time inference feasibility. To comprehensively evaluate the performance of D3PG-Light, we selected three representative continuous control DRL algorithms as baselines: Deep Deterministic Policy Gradient (DDPG), serving as the foundational actor–critic benchmark; Twin Delayed DDPG (TD3), representing state-of-the-art stability; and the original D3PG, to demonstrate the specific improvements of our lightweight modifications.

### 5.2. Performance Analysis

**Figure 5:**
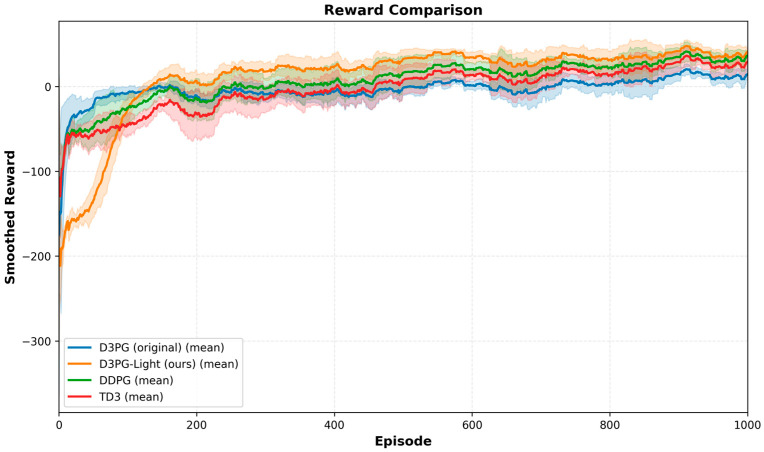
Training reward convergence curves.

Figure 5 illustrates the average reward evolution of the four compared algorithms over 1000 training episodes. All methods exhibit rapid reward improvement during the initial training phase and gradually converge to stable performance levels. In the early episodes, D3PG-Light demonstrates a more conservative learning behavior, necessitating slightly more episodes to surpass the baselines. This phenomenon is primarily attributed to two factors: the adaptive gradient stabilization (AGS) mechanism, which imposes gradient clipping to prevent parameter oscillation and ensure safe updates; and the introduction of LSTM, which requires accumulating sufficient temporal sequences to capture long-term queue dynamics accurately. Although this leads to a slower initial ascent, it effectively prevents the local optima entrapment observed in DDPG and TD3. Regarding computational cost, the entire training process of 1000 episodes takes approximately 3 h on the specified PC environment, which is acceptable for offline policy learning.

As training proceeds, D3PG-Light consistently achieves higher reward values than the baseline algorithms after approximately 200 episodes. In addition, it converges to a higher steady-state reward level with relatively smaller variance across episodes. In contrast, DDPG and TD3 exhibit more pronounced reward oscillations during convergence, which is commonly observed in continuous action ISCC environments due to value overestimation and unstable policy updates. These results indicate that the proposed lightweight design improves training stability while enabling sustained performance gains in the long-term training phase.

**Figure 6:**
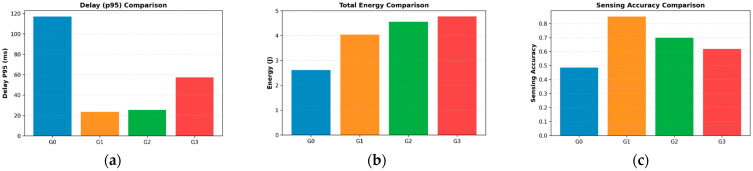
Performance comparison of different algorithms: (**a**) latency, (**b**) energy consumption, and (**c**) sensing accuracy.

To provide a comprehensive evaluation of the learned policies, Figure 6 compares the performance of different algorithms. As minimizing response time is paramount for mission-critical and safety-sensitive UAV applications, we first examine the 95th-percentile system latency. As shown in Figure 6a, D3PG-Light(G1) achieves a 95th-percentile latency of 23.6 ms, which is substantially lower than those of the baseline algorithms. Compared with DDPG(G2) and TD3(G3), the proposed method reduces tail latency by approximately 2.7 ms and 33.7 ms, respectively, while achieving an over 80% reduction relative to the original D3PG(G0). The superiority in latency performance stems primarily from the temporal modeling capability introduced by the LSTM module. Unlike the baseline algorithms that rely solely on the current state snapshot, D3PG-Light can capture the historical evolution trends of task queues. This enables the agent to adopt a ‘proactive’ scheduling strategy, allocating resources to clear potential backlogs before they cause severe congestion. Additionally, the IFF module effectively decouples queue states from channel variations, allowing the policy to precisely balance bandwidth and computing power under complex coupling constraints.

Figure 6b compares the energy consumption of different algorithms. D3PG-Light consumes slightly more energy than the resource-conservative D3PG (G0), as it supports more aggressive task processing to reduce latency. However, it remains significantly more energy-efficient than DDPG (G2) and TD3 (G3). This result demonstrates that D3PG-Light achieves a favorable balance between energy consumption and system performance, where notable latency improvements are obtained at a reasonable energy cost.

Beyond mere speed, the ultimate utility of an ISCC platform depends on its sensing effectiveness. The sensing accuracy comparison is presented in Figure 6c. D3PG-Light achieves the highest average sensing accuracy of approximately 0.85, outperforming all baseline algorithms. This result verifies the effectiveness of the proposed ISCC-oriented policy design, which explicitly considers the coupling between sensing and communication resources. By dynamically coordinating these resources, the learned policy prioritizes sensing quality under varying channel conditions while maintaining overall system efficiency. Specifically, the superiority in accuracy arises from the IFF module, which enables the agent to identify high-SNR windows and opportunistically allocate sensing resources to maximize the reward function. Regarding the absolute value, the score of 0.85 represents a normalized sensing utility. In strictly resource-constrained ISCC systems, achieving a utility of 0.85 while maintaining an ultra-low latency of 23.6 ms represents a highly effective Pareto-optimal trade-off. This significantly outperforms the baselines, which fail to balance these conflicting objectives, often sacrificing sensing accuracy to prevent queue overflows.

In addition to performance metrics, model complexity and inference efficiency are also critical factors for practical UAV deployment, especially under strict onboard computational and memory constraints. To this end, Table 2 compares the proposed D3PG-Light with representative baseline algorithms in terms of model parameter size and storage requirements. As shown in Table 2, D3PG-Light exhibits a significantly reduced model size, containing only 48,008 parameters, which is substantially smaller than those of DDPG, TD3, and the original D3PG. This compact architecture results in a storage footprint of less than 0.5 MB, making it more suitable for real-time inference on resource-constrained UAV platforms.

Importantly, this reduction in model complexity does not come at the cost of performance degradation. Combined with the results in Figure 5 and Figure 6, D3PG-Light demonstrates that a lightweight design can simultaneously achieve stable training, low latency, and high sensing accuracy. These results validate the effectiveness of the proposed lightweight architecture and justify its deployment-oriented design philosophy.

### 5.3. Ablation Study

To further validate the effectiveness of the key modules within the proposed D3PG-Light framework, ablation studies were conducted on the complete model and several simplified variants. Specifically, the temporal modeling module (LSTM), the interaction feature fusion (IFF) module, and the adaptive gradient stabilization (AGS) mechanism were systematically removed. Comparative analyses were then performed under identical simulation environments and training parameter configurations.

**Figure 7:**
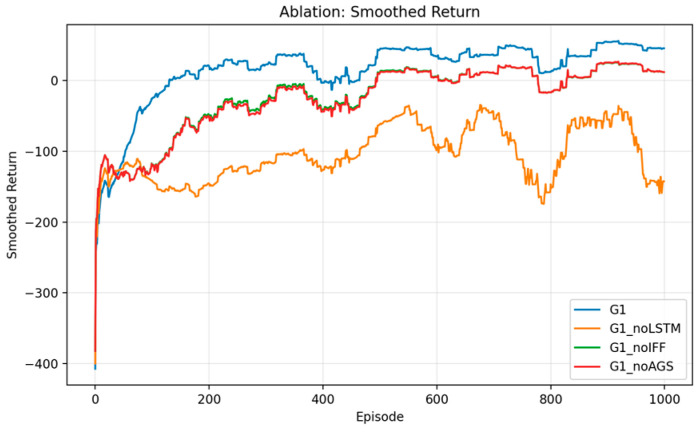
Ablation study on training reward convergence.

Regarding the training process, as illustrated in Figure 7, the complete model exhibits superior performance in terms of convergence rate, final reward level, and training stability. Although the model remains convergent after the removal of AGS, reward fluctuations increase significantly during training. This indicates that the gradient stabilization mechanism plays a crucial role in suppressing policy oscillation and enhancing training robustness. In contrast, excluding the IFF module results in a slower convergence rate and a lower final reward. This demonstrates that structured modeling of channel features and queue states in the ISCC scenario contributes to improving the policy’s capability to represent heterogeneous state information. Notably, upon the removal of the LSTM module, the training process shows significant degradation in the later stages, with rewards maintaining a persistently low level. This reflects that a lack of temporal modeling severely impairs the agent’s adaptability to the dynamic environment.

**Figure 8:**
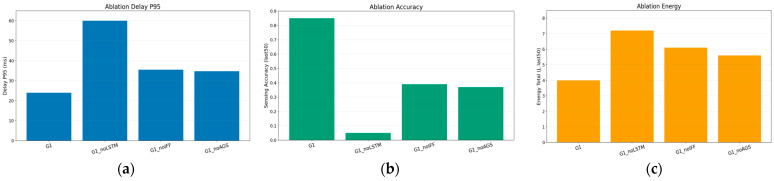
Ablation results on system performance metrics: (**a**) 95th-percentile latency; (**b**) sensing accuracy; (**c**) energy consumption.

In terms of system performance, Figure 8 presents the comparison of detailed metrics across different ablation settings. The latency performance under different ablation settings is illustrated in Figure 8a. The complete model achieves the lowest 95th-percentile latency, indicating its strong capability in suppressing tail delay. In contrast, removing the LSTM module results in a dramatic degradation of latency performance, with the tail delay exceeding 60 ms. This phenomenon confirms that temporal modeling is essential for capturing queue dynamics and maintaining system stability in time-varying ISCC environments. This observation aligns with recent findings in [10,17], which highlight that memory-based mechanisms are critical for resolving state ambiguity in highly dynamic UAV networks.

Figure 8b compares the sensing accuracy achieved by different ablation variants. The complete model consistently attains the highest sensing accuracy, demonstrating the effectiveness of the proposed ISCC-oriented design. In contrast, removing the IFF module causes a significant drop in sensing accuracy, with the average value reduced by nearly 50 percent. This observation highlights that directly feeding raw state variables into the policy network is insufficient to capture the complex coupling between communication and sensing resources.

The energy consumption comparison is shown in Figure 8c. The complete model incurs a slightly higher energy cost compared to the ablated variants. However, this increase is relatively modest and represents a reasonable trade-off. The lower energy consumption observed in ablated models, particularly the variant without the LSTM module, is achieved at the expense of severe latency degradation and reduced sensing accuracy.

Synthesizing the above results, it can be concluded that the LSTM module serves as the cornerstone for ensuring system stability and suppressing tail latency, the IFF module is fundamental for capturing the coupling between sensing and communication resources, and the AGS mechanism further enhances training robustness and performance consistency. The complete D3PG-Light framework successfully integrates these components, achieving balanced optimization across latency, energy consumption, and sensing accuracy in dynamic UAV-assisted ISCC systems.

### 5.4. Sensitivity Analysis and Reward Mechanism Validation

To further evaluate the robustness of the proposed reward formulation, a sensitivity analysis was conducted by independently varying the weight coefficients associated with latency ($w_{d}$), sensing accuracy ($w_{a}$), and energy consumption ($w_{e}$). The baseline configuration is set to $w_{a}=0.2$, $w_{e}=0.1$, and $w_{d}=1.0$. Performance metrics are computed using the median of the final 200 evaluation steps to reflect steady-state behavior.

As shown in Table 3, increasing the latency weight from 1.0 to 1.2 reduces P95 from 24.54 ms to 21.14 ms, corresponding to a 13.8% reduction in tail latency. This improvement is achieved with a moderate increase in energy consumption from 4.084 J to 4.764 J, while sensing accuracy remains nearly unchanged. Conversely, reducing $w_{d}$ to 0.8 leads to a noticeable increase in P95 and slight accuracy degradation. These results confirm that the scheduling policy responds consistently to delay prioritization and that latency can be effectively controlled through reward adjustment.

Adjusting the sensing weight influences the accuracy–latency trade-off. When $w_{a}$ is increased to 0.24, sensing accuracy improves from 0.8506 to 0.8664. However, this improvement is accompanied by an increase in tail latency and higher energy consumption. In contrast, reducing $w_{a}$ to 0.16 lowers accuracy to 0.8198 while slightly improving latency relative to the high-accuracy setting. This behavior demonstrates that the proposed reward structure enables controllable prioritization of sensing performance when required.

The energy weight governs the energy–performance balance. Increasing $w_{e}$ to 0.12 reduces average energy consumption from 4.084 J to 3.913 J but results in higher latency and noticeable accuracy degradation. Conversely, decreasing $w_{e}$ to 0.08 allows the system to utilize more energy and achieve improved tail latency. This monotonic trend indicates that the framework exhibits predictable and interpretable energy–latency trade-offs rather than unstable fluctuations.

Across all weight variations, the system behavior follows consistent multi-objective trade-off patterns without abrupt oscillations. Each objective responds directionally to its corresponding weight adjustment, and no configuration results in catastrophic instability. These findings demonstrate that the reward formulation is neither overly sensitive nor arbitrarily tuned. Instead, it provides a stable and interpretable mechanism for balancing latency, sensing accuracy, and energy consumption in dynamic UAV-ISCC environments. Overall, the sensitivity analysis confirms that D3PG-Light achieves controllable and robust multi-objective optimization, supporting the practical applicability of the proposed reward design.

## 6. Conclusions

This paper addresses the multi-resource collaborative scheduling problem in low-altitude Unmanned Aerial Vehicle (UAV) Integrated Sensing, Communication, and Computing (ISCC) systems, investigating methods to achieve low-latency and high-stability decision-making under realistic air-to-ground channels and dynamic task conditions. To address issues in existing research, such as idealized environmental modeling, instability in reinforcement learning training, and insufficient multi-objective synergy, a lightweight and highly stable deep reinforcement learning (DRL) scheduling refinement, named D3PG-Light, is presented. Building upon the classic DDPG architecture, this method introduces a tailored interaction feature fusion mechanism, an adaptive gradient stabilization mechanism, and a temporal modeling module. These enhancements improve convergence stability and tail-latency performance while keeping the model size below 0.5 MB. In particular, the incorporation of twin critics and clipped double Q-learning effectively mitigates overestimation and suppresses convergence oscillation in high-dimensional continuous action spaces.

In terms of system modeling, a unified UAV-ISCC environmental model is constructed. This model characterizes channel time-varying properties based on real air–ground channel data and jointly models the queue dynamics and latency evolution processes for communication, computing, and sensing tasks, thereby providing a high-fidelity environment for agent learning that closely mimics actual deployment scenarios. Regarding algorithm design, issues of convergence oscillation and performance degradation, which are prone to occur in reinforcement learning within high-dimensional continuous action spaces, are effectively mitigated through structured state feature processing, gradient stability constraints, and temporal dependency modeling.

Experimental results verify that D3PG-Light consistently outperforms DDPG, TD3, and the original D3PG in key performance metrics. Specifically, the proposed method suppresses long-tail delay, reducing the 95th-percentile system latency from over 100 ms to approximately 24 ms, while achieving higher steady-state reward levels with reduced variance. The parameter scale is limited to fewer than 50k parameters, corresponding to a model size below 0.5 MB, which enables efficient inference on embedded UAV platforms. At the same time, the lightweight architecture limits the model size to below 0.5 MB, enabling efficient inference on embedded UAV platforms and confirming its practical deployability.

Ablation studies further indicate that the temporal modeling module plays a decisive role in guaranteeing system stability and suppressing long-tail latency, while the feature fusion and gradient stabilization mechanisms serve important auxiliary functions in elevating the performance ceiling and enhancing robustness. These results suggest that in dynamic ISCC scenarios, decision-making policies relying solely on static state mapping struggle to meet practical requirements; the introduction of temporal dependencies and stabilization designs is key to realizing highly reliable scheduling. Furthermore, sensitivity analysis confirms that the reward formulation enables controllable and interpretable trade-offs among latency, sensing accuracy, and energy consumption, without introducing unstable performance oscillations.

Although this work has achieved progress in model stability and environmental realism, there remains room for further expansion. Future research can be conducted in the following directions: First is by extending the proposed method to multi-UAV collaborative ISCC scenarios to investigate resource competition and cooperation mechanisms among multi-agents. Second is introducing more refined sensing models and multi-modal information to improve modeling capabilities for complex sensing tasks. Third is combining model compression with online fine-tuning techniques to further enhance the algorithm’s adaptability and long-term operational performance on resource-constrained UAV platforms. These directions will contribute to promoting the practical implementation of ISCC theory and intelligent UAV control in 6G low-altitude networks.

## Figures and Tables

**Table 1:** Simulation parameters and system settings.

Parameter	Symbol	Value
Total System Bandwidth	$B_{total}$	5 MHz
Minimum Bandwidth Floor	$B_{min}$	10 kHz
Sensing Resource Coupling Factor	k	0.15
Sensing SNR Gain Factor	η	0.8
Noise Power Spectral Density	$N_{0}$	−174 dBm/Hz
Max Computing Capacity	$C_{max}$	5 GFLOPs
Task Arrival Rate (Data)	$\lambda_{b}$	2 Mbps
Task Arrival Rate (Comp)	$\lambda_{c}$	0.8 GFLOPs
Time Slot Duration	$\Delta_{t}$	10 ms
Discount Factor	γ	0.99
Actor Learning Rate	$lr_{actor}$	$1\times 10^{-5}$
Critic Learning Rate	$lr_{critic}$	$1\times 10^{-6}$
Replay Buffer Size	D	100,000
Update Rate	τ	0.005

**Table 2:** Model complexity.

Algorithm	Model Parameters	Storage Size (MB)
D3PG-Light	48,008	0.4578
D3PG	178,184	1.6993
DDPG	281,096	2.6807
TD3	421,898	4.0235

**Table 3:** Sensitivity analysis of reward weights (tail-200 median statistics).

Weight Setting	P95 (ms)	Accuracy	Energy (J)
Baseline	24.54	0.8506	4.084
$w_{d}=0.8$	28.46	0.8404	4.628
$w_{d}=1.2$	21.14	0.8498	4.764
$w_{a}=0.16$	26.22	0.8198	4.134
$w_{a}=0.24$	28.81	0.8664	4.666
$w_{e}=0.08$	21.14	0.8503	4.478
$w_{e}=0.12$	29.41	0.7497	3.913

## Data Availability

The data presented in this study are available on request from the corresponding author. The data are not publicly available due to ongoing patent-related considerations and file-size limitations associated with the measured channel dataset.
